# Persistent gender gaps in weekend leisure from ages 10 to 17: A multi‐national time diary analysis since 2010

**DOI:** 10.1111/jora.70268

**Published:** 2026-09-18

**Authors:** Grace Chang, Leena Bhattacharya, Man‐Yee Kan

**Affiliations:** ^1^ Department of Sociology University of Oxford Oxford UK; ^2^ WageIndicator Foundation Amsterdam the Netherlands; ^3^ Department of Econometrics and OR Tilburg University Tilburg the Netherlands

**Keywords:** adolescence, gender, leisure, time use

## Abstract

Gender inequalities in leisure time are understudied comparatively in East Asia and the Global South. Most studies often analyze the average of weekend and weekday activities, underestimating the gaps during the days when adolescents have the most discretionary time. Using time diary data from seven countries in the West, Asia, and Africa, we examine gender gaps in weekend leisure time across early and late adolescence (10–17 years). Universally, girls engage in less leisure than boys, driven by low engagement in sports/exercise, especially for girls in Asian countries. There is little evidence that more gender equal countries have smaller gender gaps. Girls and boys engage in gender‐typical activities; boys engage in computer/internet use more, while girls engage more in conversation. Engagement in sports/exercise falls between early and late adolescence, and the gaps persist across ages in all countries except Belgium. Any narrowing in gender gaps across age is through boys lowering their engagement in sports/exercise or increasing it through conversation, with only a decline in leisure seen in girls. Overall, gendered inequality in leisure emerges early and persists throughout adolescence.

## GENDER INEQUALITIES IN LEISURE DURING ADOLESCENCE

Adolescents' leisure time is an important element in their learning and is positively related to their developmental outcomes and well‐being (Cha & Yoo, 2024; Wanka et al., 2025). Studies across several countries show that leisure is not distributed equally, even in childhood. From the age of 10, boys and girls spend their leisure time differently; boys tend to do more sports/exercise and play video games, while girls tend to socialize (on social media) and have more creative pastimes (Chang & Kan, 2025; Gracia et al., 2020). In this paper, gender is understood not only as a categorical distinction between boys and girls, but also as a social system through which adolescents learn and enact culturally specific expectations of masculinity and femininity (West & Zimmerman, 1987; Paechter, 2006). These gender differences can lead to inequalities in future outcomes, such as lower subjective well‐being (Cha & Yoo, 2024). However, there is still limited evidence about non‐Western country contexts, and gender time use inequalities across early and late adolescence. Leisure gender gaps also tend to be underestimated, as studies usually focus on weekdays, or the average time across weekday and weekend activities. Weekend activities show greater gender variation since adolescents' time is not structured by the school day and is dependent instead on their families' schedules and resources (see e.g., Hook (2017) for adult women). Tasks that are more easily deferred (e.g., exercise) are more likely to be performed on the weekend.

We examine the universalization or divergences in gendered adolescents' weekend leisure time, using time diaries across seven countries in Asia, Africa, and Western contexts: Belgium, the United Kingdom (UK), the United States (US), Japan, South Korea—hereafter Korea, South Africa, and India. We examine this across countries and variations between early and late adolescence. Our contributions are two‐fold: First, we provide country‐specific analysis across seven countries to examine differences in gendered leisure time from the 2010s, when technology became central to leisure, including less‐studied contexts such as Japan, South Africa, and India. Second, we examine the intersection of gender and age to examine how these gaps evolve across adolescence.

## DEFINITIONS AND COUNTRY COMPARISONS

We focus on leisure activity categories that standardly show gendered patterns. We define “general leisure” to capture varied forms of leisure, including reading, outdoor leisure, indoor leisure, computer/internet use, and conversations. We then distinguish “general leisure” from sport/exercise and TV/radio. Physical and media leisure are studied separately since the 2010s; there have been patterns and concerns about adolescent sedentary behavior, technology, and screen time (Cha & Yoo, 2024; Rees, 2017; Román & Gracia, 2022), especially for girls in later adolescence (Felez‐Nobrega et al., 2020; Guo et al., 2024). Lastly, due to data limitations, we analyze computer/internet use and conversations as a separate subset from “general leisure.” Conversation is often highlighted to be performed mostly by girls, and this activity tends to crowd out leisure activity as girls get older (McHale et al., 2012; Richards & Larson, 1989).

Table 1 compares countries which vary in access to and norms around leisure. Western societies such as Belgium, the UK, and the US are individualistic societies with a preference for leisure, with low gender inequality index (GII, <0.2), average levels of safety (50% feel safe at being in public spaces), and high access to computers/internet (over 90%). East Asian societies such as Japan and Korea exhibit similarly low GII (about 0.1 or lower), internet accessibility (over 90%), and high safety levels (around 70%), but their cultures are rooted in Confucianist values that emphasize educational activities and family duties, focusing more time on education and less on leisure (see e.g., Ahn & Yoo, 2022; Chang & Kan, 2025; Román & Gracia, 2022). However, Japan and Korea also differ. Japan has stronger health promotion plans (Shibaike et al., 2002) thus adolescents may engage more in physical activity.

**TABLE 1:** Macro‐level indices of countries.

Country	Years	Gender Inequality Index (GII)	Gallup Safety Index	Internet access
Belgium	2013	0.073	50.8	82.2%
United Kingdom	2014	0.143	52.6	91.6%
United States	2009–2023	0.260–0.169	50.7	71.0% in 2009; 93.5% in 2023
Japan	2011	0.117	77.4	79.1%
	2016	0.104		93.2%
South Korea	2014	0.070	75.1	87.6%
	2019	0.051		96.2%
India	2024	0.403	55.4	60.3%
South Africa	2010	0.441	24.6	24.0%

*Note*: Years reported are based on country years used in this manuscript. Sources: GII: UNDP Human Development Report via Our World in Data. GII using the UNDP index measures it based on reproductive health, maternal mortality, adolescent birth rates, parliamentary representation, education, and labor‐market participation. Safety: Gallup (2014–2023 scores where percentage refers to percentage of sample of the survey who feel safe being in public spaces after dark) See e.g., Gallup (2025). Computer/internet accessibility: https://data.worldbank.org/indicator/IT.NET.USER.ZS.

Comparatively less studied are patrilineal societies such as India and South Africa, with greater GII (0.4). Children in both countries engage in significant domestic work, and girls primarily shoulder the burden (Ladusingh, 2017; Statistics of South Africa, 2021). In India, the public environment tends to be “male‐dominated” where women are expected to stay at home (Ladusingh, 2017). In South Africa, public safety concerns are highest (24.6% feel safe being in public after dark) with the lowest internet penetration (24%) and significant inequalities due to ethnic and linguistic differences (Wushe et al., 2014).

## HYPOTHESES AND MECHANISMS

We make the following hypotheses about the comparative difference in adolescents' overall levels of engagement in leisure activities and the gaps in these activities.

“General leisure” is likely higher in Western societies, lowest in Japan and Korea, and in the middle for India and South Africa.

Computer/internet use is likely highest in Western societies, and physical activity is lowest in all other societies.

Gender gaps in leisure could be explained by gender socialization theory (Bandura, 1977), which posits that children learn and internalize gender‐typed behaviors or attitudes through parental modeling, sibling or peer influence. If gender norms dictate that there are large gender divisions of paid and unpaid labor in the household, this will be reflected in adolescents' activities. Pal (2026) found that in India, gender disparity in adolescent activities follows traditional gender norms reflected by adults. We further posit that countries with lower GII reflecting greater adult gender equality, will exhibit smaller gender gaps in adolescents' leisure time than countries with higher levels of GII (e.g., India and South Africa).

Gender‐typical leisure activities: Boys are more likely to spend time on physical activity, while girls are more likely to spend time on conversations.

Gender gaps in leisure activities are likely narrower in countries where the GII is lower.

Leisure generally declines with age as responsibilities increase (Hilbrecht et al., 2008). Studies show that physical activity declines from early to late adolescence, especially due to a decrease in free play and greater time in academic responsibilities (Hilbrecht et al., 2008; van Mechelen et al., 2000; Olds et al., 2009). There is mixed evidence about the gender gaps across age, where some studies find a narrowing in gender gaps (Hilbrecht et al., 2008; van Mechelen et al., 2000) and others find a widening by age 17 (Olds et al., 2009).

For other leisure activities, studies posit that the types of engagement follow the “gender intensification hypothesis (Hill & Lnch, 1983),” that is, increasing pressure to conform to traditional gender roles during and after puberty. This is also in line with “doing gender,” a theory that views gender as performative through everyday interactions in the household or environment (West & Zimmerman, 1987), reinforcing norms that reward or prevent certain male‐ or female‐oriented traits. For example, boys are drawn to competitive gaming or sports (e.g., Hilbrecht et al., 2008), whereas girls are steered towards aesthetic or relational hobbies (e.g., McHale et al., 2004). Studies, based in the Global North, show that gender may be more performative after puberty, where gender‐typical activities emerge during middle adolescence (15–17), with girls spending less time in sports and more time socializing (McHale et al., 2004; Richards & Larson, 1989; Timmer et al., 1985). In India and South Africa, there may be greater gendered polarization, such as intrahousehold resource allocations where parents or guardians reallocate investments to their children based on their age, gender, and ethnicity/linguistics. Therefore, we expect that adolescents are likely to perform more gender‐typical activities from early to late adolescence, but gender gaps may widen further in India and South Africa as girls take on greater unpaid work responsibilities compared to boys. We hypothesize:

From early to late adolescence, gender‐typical leisure time rises, but sports/exercise for boys and girls declines.

Gender gaps may narrow with age in all countries, except for India and South Africa, where gender gaps may widen.

## DATA AND METHODS

### Data and analytic samples

We use weekend‐only time diary data available for adolescents aged 10–17 years from 2010 onwards in the following data sources: the Multinational Time Use Study (MTUS) diaries (Fisher et al., 2022), the Japan Survey of Time Use and Leisure Activities, the Korean Time Use Survey, and the Indian Time Use Survey (ITUS). See Appendix Table A1 for details on sampling and number of diaries.

The time diaries are collected at the household level. We cap at age 17 because those aged 18 and above may have moved out for education, whereas younger children might be reliant on parents. We keep Belgium, the UK, the US, and South Africa from the MTUS, as these were the only countries that had a large enough sample of weekend diaries for adolescents since the 2010s. We include Japan, Korea, and India from their respective time diaries, providing us a total of 58,570 adolescent weekend diary‐year entries.

Appendix Table A2 presents leisure time in India by day of the week, since ITUS has data for each day. Average time spent on TV/radio, sports/exercise, and overall leisure is broadly similar across weekdays but is significantly different on Saturdays and Sundays. This reinforces our focus on weekend leisure when examining gender gaps.

### Measurements and Methods

#### Dependent variables

Leisure activities are captured from time diaries, which require the respondent to answer what they did from 4:00 am to 4:00 am the next day, in 10‐ or 15‐min slots, depending on the country. We calculate the total minutes spent in the following categories:

1. “General leisure” which combines reading, gardening, pet care, leisure outside the home (movies/theater), “other” forms of leisure such as having conversations, games, art, indoor leisure, hobbies, and relaxing, *and* computer/internet use
2. Sports/exercise
3. TV/radio
4. Computer/internet‐use (not observed for South Africa, Japan, and India)
5. Conversation (not observed for South Africa and Japan)

Appendix Table A3 details the activity definitions. We group computer/internet‐use and conversation under “general leisure” because, in a subset of countries, these activities cannot be distinguished from other forms of leisure due to differences in data collection. Where data permit, we analyze (4) and (5) separately; however, they are also included within the broader category of general leisure. Sports/exercise and TV/radio are treated as distinct leisure activities.

#### Independent Variables

Our main variable of interest is gender, a binary variable (1 = girl, 0 = boy), and age consists of ages 10–17. The country variables we use are Belgium (BE) 2013, South Africa (ZA) 2010, the United Kingdom (UK) 2014, the United States (US) 2009–2019 (excluding Covid‐19 lockdown years), Japan (JP) 2011 and 2016, South Korea (KR) 2014 and 2019, and India (IN) 2024.

#### Covariates

All models include controls for the adolescent, household, and survey characteristics, harmonized across the countries. Household income is categorized as the lowest 25%, middle 50%, and highest 25% within the country. Household size is a continuous measure of all household members. We control for the number of children (0–17 years) in the household, and whether the child is the youngest in the household (1 = Yes, 0 = No). Lastly, we control for the sector of residence (1 = urban/semi‐urban, 0 = rural/semi‐rural) and survey year, ranging from 2010 to 2024, if the country has multiple survey years and if sector data is available. We use case‐wise deletion for missing information. Each analysis is conducted within country, and thus weights are not applied. For India, we show two robustness checks: (1) controlling for the state of residence to account for within‐state variation and (2) controlling for religion and caste, owing to the literature on how social identity can shape the time allocations of Indian adolescents (Pal, 2026).

To answer H1 and H2, we run *t*‐tests to examine the differences in means of time spent in each of these leisure activities. We use Ordinary Least Squares (OLS) to examine gender gaps by country. For each adolescent in each country, we estimate for each of the five leisure categories:

$$L=\alpha +\beta_{1}G+\beta_{2}I+\beta_{3}X+\varepsilon$$

where *L* represent leisure activities, G is the gender, *I* is household income, and *X* are all other covariates as listed earlier. OLS captures the average time spent, including participation levels, in each leisure activity. Each country's standard errors are clustered at the adolescent‐diary level, as there are sometimes multiple diaries per adolescent. India has only one diary per adolescent, thus estimates are clustered at the state level to address within‐state correlations in error terms, but results are robust even at alternate district‐level clusters. Average marginal effects from Tobit models which censor zeroes (exclude participation) are reported in Appendix Table A5 and show similar estimates.

To answer H3 on gender gaps across age, we conduct heterogeneity analyses by interacting age with gender gaps and report the marginal effects of these predicted estimates. As a sensitivity check, we test for variation across sectors for countries that have these data (i.e., the UK, US, ZA, and IN) to proxy for access to leisure activities.

Table 2 shows the average characteristics of the sample across countries. There is an equal split in gender composition and average age, except for the US, where adolescents are slightly older (age 16 on average). Household sizes average at 2–3 across all countries except India (5). There is roughly an equal split of sample adolescents being the youngest in the household, except for South Africa and Japan where the majority are the youngest. Household income is broadly distributed in the middle 50%, except for South Africa, where there is a more even distribution between the lowest 25% and the middle 50%. Lastly, samples are primarily urban in Belgium, the UK, the US, and South Korea, with greater sector variation in South Africa, Japan, and India.

**TABLE 2:** Average characteristics by country.

	BE (2013)	UK (2014)	US (2009–2023)	JP (2011, 2016)	KR (2014, 2019)	ZA (2010)	IN (2024)
Age	13.6 (2.2)	13.4 (2.3)	16.1 (0.8)	14.5 (2.5)	13.5 (2.3)	13.5 (2.3)	13.5 (2.3)
Household size	2.3 (0.6)	2.3 (0.6)	2.3 (0.6)	2.4 (0.5)	2.2 (0.5)	2.5 (0.6)	5.0 (1.8)
Gender							
Boys	47%	48%	52%	51%	51%	51%	53%
Girls	53%	52%	48%	48%	48%	49%	47%
Youngest in the household							
Yes	56%	56%	56%	35%	45%	74%	52%
No	44%	44%	44%	65%	55%	26%	48%
Household income							
Lowest 25%	22%	20%	16%	20%	10%	44%	27%
Middle 50%	41%	53%	44%	47%	51%	42%	53%
Highest 25%	37%	27%	40%	32%	38%	13%	20%
Locality							
Rural/semi‐rural	N/A	N/A	16%	69%	3%	54%	67%
Urban/semi‐urban	N/A	N/A	84%	31%	97%	46%	33%
Total diaries	536	776	2198	32,759	4308	1574	15,589

## WHAT ARE THE DIFFERENCES IN ADOLESCENT WEEKEND LEISURE TIME ACROSS COUNTRIES?

Adolescents spend between 3 and 6 h on weekend general leisure (see Appendix Table A4). General leisure is highest for Indian adolescents (about 5 h), and lowest in South Africa (about 3 h). This is driven by more than an hour of conversation and 30 min of “doing nothing” in India, which are significantly lower in the other countries. This does not support H1a, that general leisure is highest in Western societies and lowest in Japan and Korea. Rather, it is highest in India, and general leisure could, in fact, be inflating time spent on very low‐resource activities such as “doing nothing” in India. Overall, boys spend more time on general leisure than girls, except for Belgium, where the gaps are the narrowest.

**FIGURE 1:**
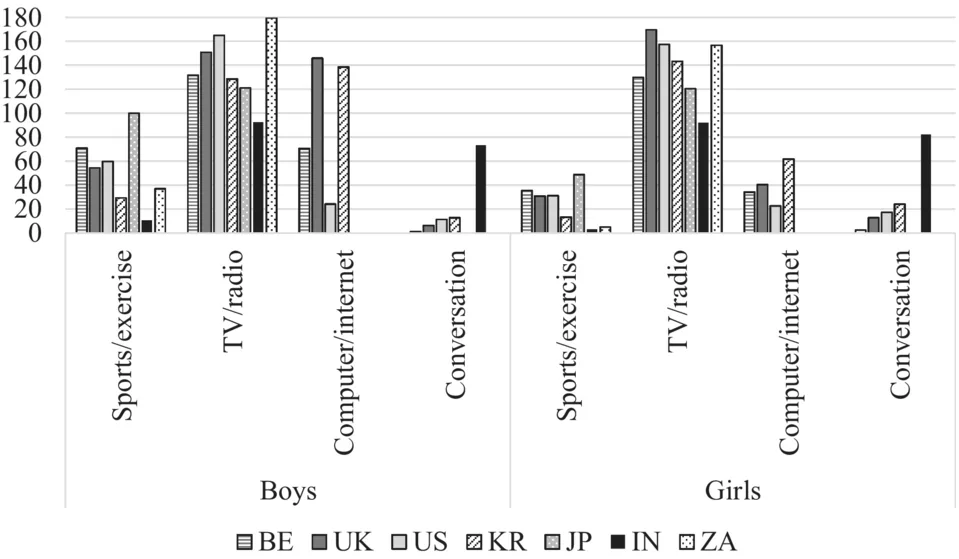
Minutes spent on weekend leisure activities across countries by gender. Data on computer/internet use is unavailable for South Africa (ZA), Japan (JP), and India (IN), and data on conversation is unavailable for ZA and JP.

Figure 1 documents the overall time spent on the remaining four leisure categories across seven countries, by gender. We exclude general leisure to allow better visibility.

Adolescents spend the majority of their time on TV/radio, with the highest for South African boys (180 min) and the lowest in Asia. Sports/exercise is lowest in India, Korea, and South Africa compared to the other countries. For boys, this figure is under 10 min in India, 29 min in Korea, and 37 min in South Africa. Boys in Japan spend the most time on sports/exercise, and boys in Belgium, the UK, and the US spend about 60–80 min. Notably, girls spend about half or less than half the amount boys spend on sports/exercise, and it is particularly low in India (3 min) and South Africa (5 min). This corroborates the literature on the high sedentary behavior of adolescents in the 21st century (Guthold et al., 2020).

For countries with computer/internet‐use information, boys in the UK and Korea spend the most time on it (about 140 min), followed by Belgium (70 min) and the US (24 min). Girls spend about half the time boys do, except in the US, where levels are approximately equal.

Lastly, the amount of time spent on conversation (where data is available) is generally low, but remarkably high in India (over an hour for both boys and girls).

There is partial support for H1b that screen time is highest in Western societies and physical activity is lowest in others. Screen time is highest in the UK and Korea, which may be driven by a “digital culture” such as gender preferences around the e‐gaming industry. While physical activity is lower in non‐Western societies, it is uniquely high for Japanese boys. This may be driven by Japan's government initiatives to improve sports among youth, more helpful for boys than girls.

## GENDER GAPS IN LEISURE

Table 3 reports the gender gaps coefficients separately for each leisure activity by country. Girls spend significantly less time on general leisure across all countries, except for Belgium and South Africa. The gaps are largest in the UK (75 min) and the US (55 min), and smallest in Belgium (7 min). Gender gaps are smallest in TV/radio, favoring girls in the UK (18 min) and Korea (15 min) but favoring boys in the US (−10 min) and South Africa (−26 min).

**TABLE 3:** Coefficient of gendered weekend leisure by country.

	Belgium	United Kingdom	United States	Japan	South Korea	South Africa	India
General leisure	−7.45 (14.47)	−75.18*** (11.78)	−54.65* (4.91)	−36.41*** (2.31)	−49.69*** (4.08)	−11.44 (7.73)	−43.01*** (3.70)
Sports/Exercise	−33.66*** (8.42)	−23.99*** (5.43)	−28.55 (6.28)	−50.55*** (1.66)	−16.21*** (1.46)	−31.40*** (3.38)	−6.76*** (1.05)
TV/Radio	−0.97 (9.46)	17.80* (9.68)	−9.87** (0.38)	−0.62 (1.58)	14.79*** (3.29)	−26.59*** (6.98)	0.76 (1.44)
Computer/internet‐use	−36.65*** (9.23)	−105.50*** (9.03)	−2.82* (0.28)	N/A	−76.01*** (3.17)	N/A	N/A
Conversation	1.73* (1.03)	6.57*** (2.16)	5.71 (3.88)	N/A	14.79*** (3.29)	N/A	8.35*** (2.25)
Observations	536	776	2198	32,759	4308	1574	15,589

*Note*: All estimates control for individual and household covariates. Year fixed effects are included for countries with multiple survey years: US, Japan, and South Korea. A robustness test for India, as the country information is largest and richest, is shown in Appendix Table A6.

* *p* < .1.

** *p* < .05.

*** *p* < .01.

Boys spend more time on sports/exercise and on computer/internet use, while girls spend more time on conversations, in all countries except the US. Hence, the general leisure gaps largely reflect boys' greater engagement in these activities (where measurable). The gender gap in sports/exercise is largest in Japan (50 min), followed by South Africa and Belgium, the UK, Korea, and India (7 min). Since girls' time spent on sports/exercise in India and South Africa is nearly negligible (3 and 5 min, respectively), the gap in South Africa is particularly stark at 31 min, possibly reflecting constraints related to female public safety.

Levels of computer/internet use in the UK and Korea are highest compared to the other countries, with the largest gender gaps. Boys in the UK spend about 105 min more on computer/internet use compared to girls, more than double the gaps in Belgium, and almost double to Korea.

Girls spend more time conversing than boys, but the gaps are small, that is, 2 min in Belgium, 7 min in the UK, 8 min in India, and 12 min in Korea. In India, recall that overall levels in conversation are highest for both girls and boys (over an hour), but still a gap remains.

We find support for H2a that girls and boys have gender‐typical leisure activities. Boys spend more time on general leisure, sports/exercise, and computer/internet use, while girls spend more time on conversations. We do not find support for H2b that more gender equal countries necessarily have smaller gender gaps. While we do find that girls and boys spend similar amounts of time on TV/radio, boys spend significantly more time on computer/internet use, particularly in the UK and South Korea, where the gaps are over an hour.

## GENDERED GAPS ACROSS AGE

**FIGURE 2:**
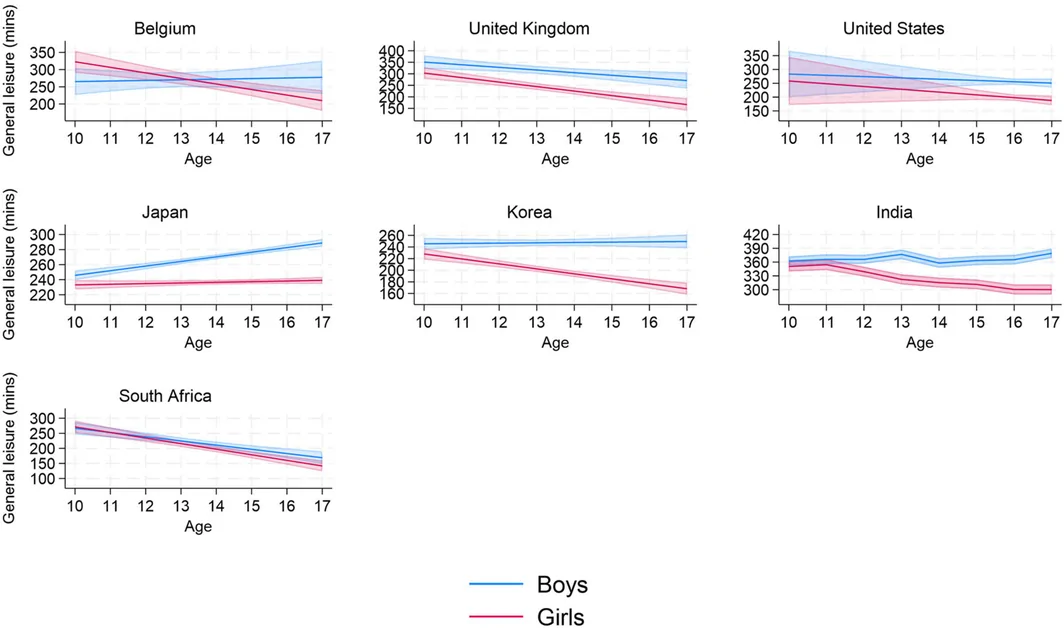
Interaction of age and gender with 95% CI for general leisure. The y‐axis is kept at different scales, as the readability would be compromised across the countries.

Figure 2 reports the marginal effects of age interacted with the gender gap for general leisure. A table of interaction coefficients is in Appendix Table A7. General leisure declines across all countries across age, except for Japan, where leisure steadily rises for boys. In Belgium, the US, and South Africa, the marginal effects of age and gender overlap across time, indicating no significant differences across ages. Notably, adolescents in the US have a higher average age of 16, and we are unable to make generalizations about their predicted age marginal effects at much younger ages (and confidence intervals are large). Gender gaps are most evident and widen across age in Japan, Korea, and India. In Japan, boys' general leisure increases with age, while girls' leisure remains flat. In Korea and India, girls' general leisure decreases by at least an hour relative to boys by age 17. Boys' general leisure remains constant across age.

**FIGURE 3:**
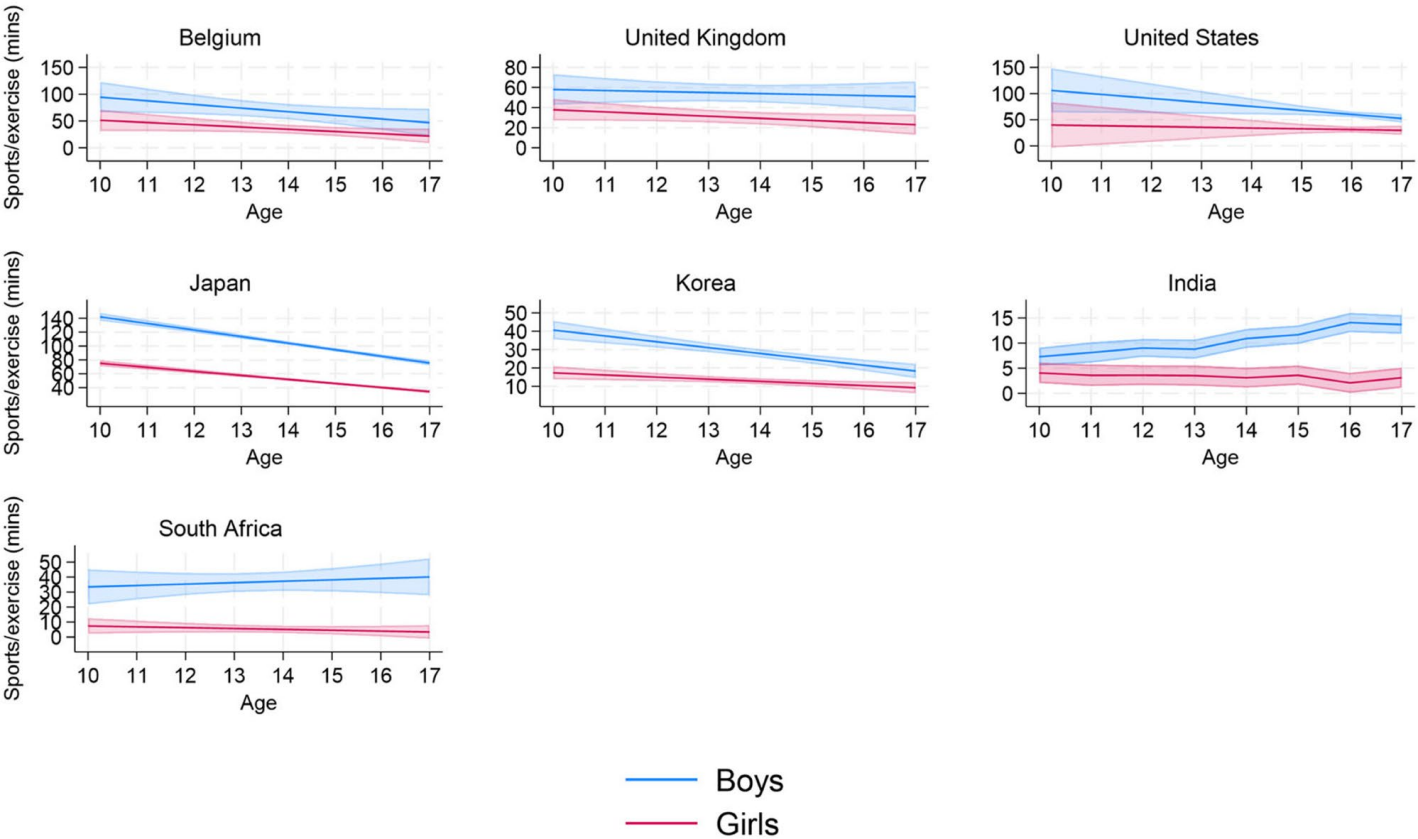
Interaction of age and gender with 95% CI for sports/exercise.

Figure 3 reports the same estimates but for sports/exercise, showing a decline across age in all countries, with significant but narrowing gender gaps. These narrowing gaps are driven by boys decreasing time in sports/exercise, except for South Africa and India, where levels remain consistent. The decline in girls' sports/exercise is steepest in Japan and Korea, where girls spend half the time on sports/exercise at age 17 compared to age 10, especially in Korea, where girls spend 13 min on average.

Figures A2, A3, A4 show a mixed narrative for the other four categories of leisure. Gender gaps in TV/radio are insignificant, except for ages 10–15 in Korea, and ages 13–17 in South Africa. TV/radio time decreases in all countries except for South African boys, who spend 40 min more than girls by age 17. Boys spend increasing time on computers/internet across all ages, with evident gender gaps in the UK and Korea, where engagement is highest. Girls and boys spend increasingly more time in conversations in the UK, Korea, and India, but with significant gender gaps only in Korea.

We find partial support for H3a. Girls spend more time on female‐oriented activities (conversations) and boys spend more time on male‐oriented activities (computer/internet use). Overall time on sports/exercise declines in all countries except for South African boys.

**FIGURE A1:**
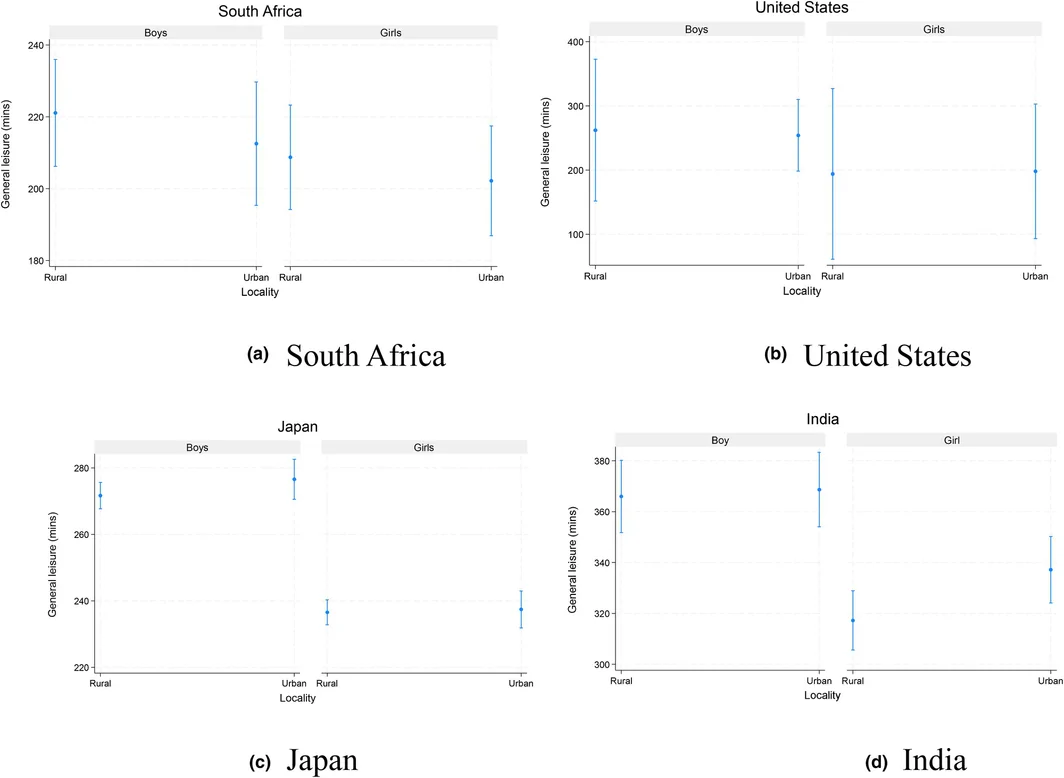
Marginal effects of predicted leisure time by sector and gender. Similar estimates were found for sports/exercise and TV/radio.

**FIGURE A2:**
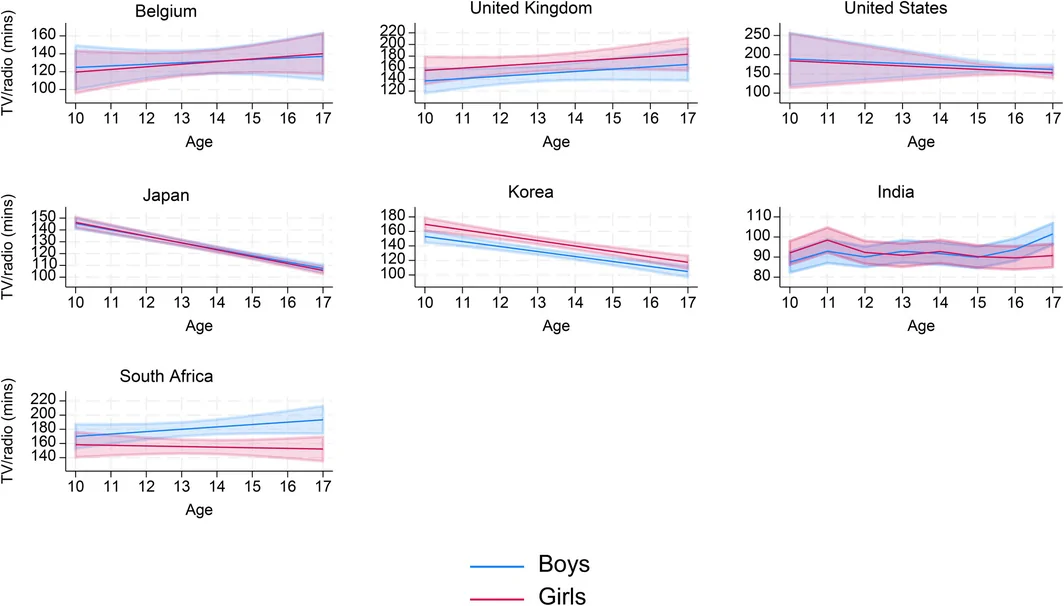
Interaction of age and gender with 95% CI for TV/radio.

**FIGURE A3:**
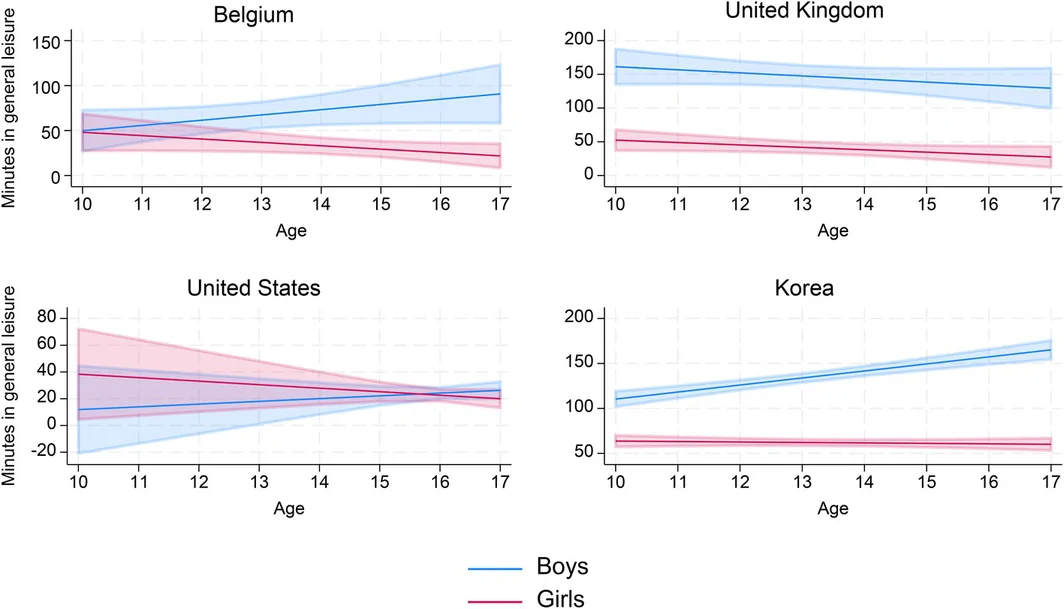
Interaction of age and gender with 95% CI for computer/internet use.

**FIGURE A4:**
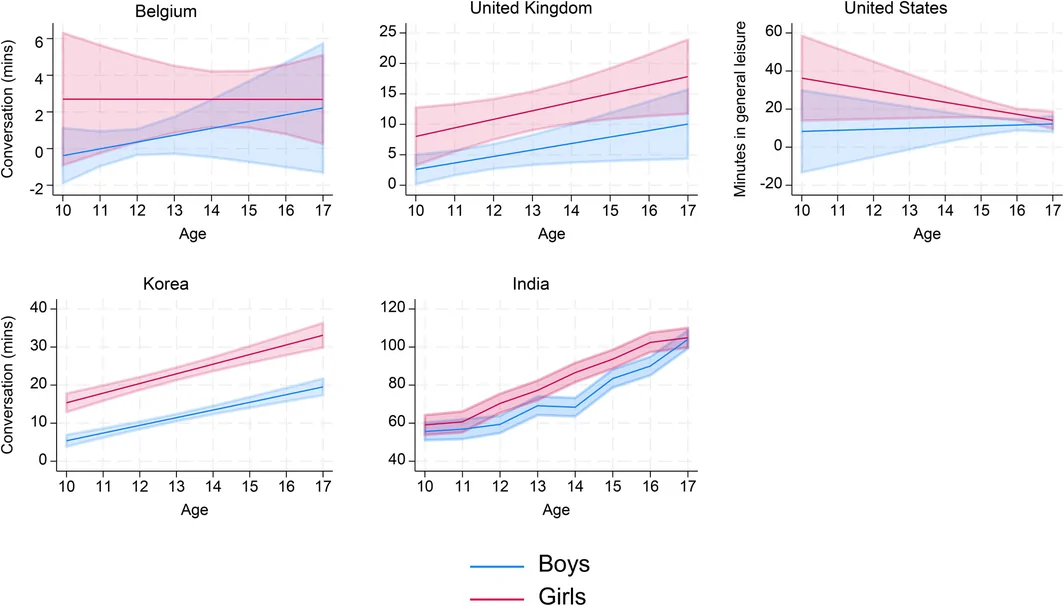
Interaction of age and gender with 95% CI for conversation.

We find mixed evidence for H3b. Some gaps widen (e.g., general leisure, computer/internet‐use), while others narrow (e.g., sports/exercise and conversations), but without a consistent pattern. Gender gaps in general leisure widen across age in India but not in South Africa. Conversely, gender gaps widen for TV/radio and sports/exercise in South Africa, but not in India. Where gaps narrow, this is primarily driven by boys reducing time in sports/exercise or increasing time in conversations. No significant sectoral (rural/urban) effects were observed for South Africa, the US, Japan, and India (Figure A1).

## DISCUSSION AND CONCLUSIONS

Striking gender inequality patterns are found across all countries, indicating universal trends: girls spend less time than boys on most forms of leisure, except for conversation, where gender gaps are small (under 12 min). Between early and late adolescence, the time spent on all leisure activities decreases for girls except for conversations, whereas patterns for boys are more variable. These findings point to a concerning, persistent gender gap in leisure: girls not only have low engagement in health‐promoting leisure, but their participation in these activities further declines between ages 10 and 17. Our findings support the gender socialization theory, suggesting an internalization of gender‐typical activities and societal expectations from young. There is partial support for the “gender intensification theory” in line with “doing gender,” as girls spend increasingly more time in conversations during adolescence. However, this theory does not explain why boys increase their time conversing and decrease their time on sports/exercise. Other country‐specific factors that may influence participation in sports/exercise remain beyond the scope of this study. Girls in India and South Africa, where compulsory schooling years are lower, might replace leisure with domestic work. For countries where school enrollment is high, this may be due to rising academic demands for both boys and girls.

We do not find consistent evidence that gender differences in leisure are driven by macro‐level factors. Greater adult gender equality does not necessarily correspond to smaller leisure gaps. In Japan, Korea, and India, the largest gaps occur in sports/exercise. In South Africa, the widest gap is in TV/radio, possibly reflecting limited leisure alternatives. The UK and Korea show the largest gender differences in computer/internet use, consistent with previous studies (Chang & Kan, 2025; Gracia et al., 2020), which may reflect a stronger male‐oriented e‐gaming or internet culture.

Our findings use cross‐sectional data, limiting our ability to draw firm conclusions about changes in time use across age. Data harmonization challenges also restrict information on computer/internet use and conversations in certain countries. The US is the only country with a higher average age, limiting the interpretation of the age and gender gap interaction in the country. Nevertheless, we demonstrate across diverse national contexts, girls experience a consistent leisure deficit relative to boys from a young age, particularly in sports/exercise– where gaps emerge early and widen with age.

We demonstrate the persistent and early emerging gender gap in leisure, particularly in physical activity, highlighting the need for context‐sensitive interventions to engage adolescents in health‐promoting activities. Increasing access to facilities, public safety, and leisure infrastructure is essential to prevent inequalities from becoming entrenched and affecting long‐term health and well‐being.

## AUTHOR CONTRIBUTIONS

**Grace Chang:** Conceptualization; investigation; writing – original draft; methodology; validation; visualization; formal analysis; project administration. **Leena Bhattacharya:** Methodology; formal analysis; validation; visualization; investigation. **Man‐Yee Kan:** Writing – review and editing; conceptualization; supervision; funding acquisition; resources.

## FUNDING INFORMATION

This work is supported by funding from the European Research Council (PI: Prof Man‐Yee Kan, Grant number: 771736) under the Horizon 2020 programme.

## CONFLICT OF INTEREST STATEMENT

The authors declare that there are no conflicts of interest.

## ETHICAL STATEMENT

Ethics approvals have been ensured by all institutes (usually the national statistics office) that collected and/or harmonized the respective time diary information, where participant details have all been anonymized. Data are restricted for Japan and South Korea, and only available to registered users for the Multinational Time Use Survey and for India.

## PATIENT CONTENT STATEMENT

Patient content statement is not applicable or needed in this study, as the data is based on household‐level information and consent was obtained by the main respondent of the survey (usually the household head).

## Data Availability

The data that support the findings of this study are available from the Multinational Time Use Survey and the Indian Time Use Survey (https://microdata.gov.in/NADA/index.php/catalog/236/study‐description), which are available to registered users. Data from the Japan Survey of Time Use and Leisure Activities and Korean Time Use Survey (http://kostat.go.kr/portal/eng/index.action), is restricted. Restrictions apply to the availability of these data, which were used under license for this study. Syntax used to harmonized the diaries can be found at https://www.gentime‐project.org/ for Japan and Korea, and in the Appendix for India. Stata syntax codes for this manuscript is available on Github at: https://github.com/gczching/Adolescent_timeuse_JRA.

## APPENDIX A

**TABLE A1:** Descriptives of sampling method for time diaries in each country.

Country	Years (*n* = total sample)	No. of weekends & weekdays	Sampling procedures
Belgium	2013 (*n* = 1225)	Weekday: 614 Weekend: 611	Household‐based national probability sample, drawn from the Belgian population/register frame and fielded to private households. Within sampled households, household members in the eligible age range completed time‐use instruments. Diary days were assigned to cover weekdays and weekend days, following HETUS‐style (Harmonized European Time Use Surveys) procedures.
South Africa	2010 (*n* = 7318)	Weekday: 5681 Weekend: 1637	Stats SA used its household‐survey master sample. The design was stratified two‐stage: first‐stage PSUs/enumeration areas selected with probability proportional to size; second‐stage dwelling units selected systematically. It covered all provinces and strata such as urban formal, urban informal, rural formal, and tribal areas. In each household, up to two eligible persons aged 10+ were selected for the diary.
United Kingdom	2014 (*n* = 1717)	Weekday: 874 Weekend: 843	Random‐probability household sample of private addresses across the UK, with fieldwork spread across the year. All eligible household members, generally age 8+, were asked to complete individual questionnaires and two diaries, usually one weekday and one weekend day.
United States	2009–2019 (*n* = 4426)	Weekday: 2207 Weekend: 2219	The American Time Use Survey sample is drawn from households that have completed their final interview in the Current Population Survey. One person age 15+ is randomly selected per household. Respondents are assigned a specific diary day, with the sample balanced so that about half report weekdays and half report weekend days; no proxy or substitute diary day is allowed.
Japan	2011 (*n* = 27,568)	Weekday: 10,699 Weekend: 16,869	Official stratified two‐stage sample. First stage: Population Census enumeration districts (ED), stratified by the 47 prefectures and selected systematically with unequal probabilities based on population. Second stage: 12 households per sampled ED selected systematically from household lists. All household members age 10+ were enumerated. Some EDs affected by earthquakes were excluded.
	2016 (*n* = 26,441)	Weekday: 9947 Weekend: 16,494	
South Korea	2014 (*n* = 6276)	Weekday: 3819 Weekend: 2457	National household probability sample based on census/sample enumeration districts. Stratified area sampling: sample districts selected first, households selected within districts, and household members age 10+ complete time diaries. Diaries are assigned across days of week and field periods to represent usual weekly time use.
	2019 (*n* = 4570)	Weekday: 2719 Weekend: 1851	
India	2024 (*n* = 62,815)	Mon: 9374 Tues: 9601 Wed: 9353 Thu: 9386 Fri: 9511 Sat: 8840 Sun: 6750	National household probability survey using a stratified multi‐stage design. In rural areas, villages are the first‐stage units; in urban areas, Urban Frame Survey blocks are the first‐stage units. Households are selected within sampled first‐stage units, with an intermediate hamlet‐group/sub‐block stage where large villages or blocks require segmentation. All eligible household members, typically age 6+, complete a 24‐h time‐use diary/time‐disposition schedule. The design is intended to produce national and state/UT rural–urban estimates. Survey is face‐to‐face format. Information related to time use was collected for 30‐min blocks for a total of 165 groups of activities, as per the International Classification of Activities for Time Use Statistics 2016 classification. The respondent could report up to three activities in a given time slot, provided that they had spent at least 10 min in that activity. In the case of multiple activities in a given time slot, the respondents reported the major activity as the one which they considered to be the most important in that time slot.

*Note*: Multinational Time Use Study (MTUS) diaries (https://timeuse.org/mtus), the Japan Survey of Time Use and Leisure Activities, the Korean Time Use Survey (http://kostat.go.kr/portal/eng/index.action), and the Indian Time Use Survey (https://microdata.gov.in/NADA/index.php/catalog/236/study‐description). Syntax used to harmonized the diaries can be found at https://www.gentime‐project.org/ for Japan and Korea.

**TABLE A2:** Minutes per day on leisure activity by type of day and gender in India.

Day of week	TV/Radio			Sports			General leisure		
	Boy	Girl	Total	Boy	Girl	Total	Boy	Girl	Total
Monday	70	69	70	7.7	2.3	5.2	283	248	266
Tuesday	70	67	68	7.2	2.6	5.0	281	242	263
Wednesday	71	68	70	7.7	2.6	5.3	280	250	266
Thursday	71	70	71	6.8	2.9	4.9	286	253	270
Friday	72	69	70	7.3	2.3	4.9	288	254	271
Saturday	76	74	75	8.3	3	5.8	304	268	287
Sunday	114	115	115	13	3.8	8.8	451	396	425
Total	76	74	75	8.1	2.7	5.6	304	268	287

*Note*: Unweighted sample estimates calculated for children aged 10–17 years.

**TABLE A3:** Definitions of leisure activities.

Types of leisure	Activity codes
General leisure	MAIN35 General out of home leisure (unspecified leisure away from home) MAIN36 Attend sporting match or games MAIN37 Cinema, theater, opera, concert MAIN38 Other public event (museum, art exhibition, zoo, walking tour, library not for study) MAIN39 Restaurant, café, bar, pub MAIN40 Party, reception, social event MAIN45 Other out‐of‐doors recreation (camping, at beach, children playing outdoors) MAIN46 Gardening/forage, hunting/fishing MAIN47 Walking dogs (or other animals) MAIN27 Pet care (look after, groom) MAIN48 Receive or visit friends MAIN50 Games (social or solitary), other in‐home social MAIN51 General indoor leisure (children playing inside/unspecified) MAIN52 Artistic or musical activity MAIN53 Written correspondence MAIN54 Knit, crafts, hobbies MAIN55 Relax, think, do nothing MAIN56 Read MAIN60 Computer games alone or in groups or online MAIN61 Send email, surf internet, programming, computing
Conversation	MAIN49 Conversation
TV/Radio	MAIN57 Listen to music, audiobook MAIN58 Listen to radio MAIN59 Watch TV, DVD, including streamed content
Sports/exercise	MAIN42 General sport or exercise MAIN43 Walking (not walking dogs) MAIN44 Cycling
Computer/internet use	MAIN60 Computer games alone or in groups or online MAIN61 Send email, surf internet, programming, computing

*Note*: These activity categories were also followed for the India Time Use Survey, but are recorded according to ICATUS 2016 in the original data.

**TABLE A4:** Average minutes in weekend leisure activities across countries and by gender.

	BE	ZA	UK	US	JP	KR	IN
**General leisure**							
Boys	270.6	220.4	312.7	255.4	272.9	247.9	367.06
Girl	263.7	202.5	235.7	197.5	237.0	197.8	323.62
Gap	6.9	17.9**	77.0***	57.9***	35.9***	50.1***	43.44***
**Sports/exercise**							
Boys	70.8	36.9	54.5	59.7	99.9	29.2	10.43
Girl	35.5	4.9	30.7	31.1	48.7	13.3	3.32
Gap	35.3***	32.0***	23.8***	28.6***	51.2***	15.9***	7.11***
TV/radio							
Boys	131.6	179.7	150.8	164.9	121.1	128.6	92.43
Girl	129.9	156.6	169.4	157.3	120.5	143.3	92.02
Gap	1.7	23.1**	−18.6*	7.6	0.6	−14.7***	0.41
**Computer/internet use**							
Boys	70.6		145.7	24.2		138.5	
Girl	34.1		40.4	22.7		61.7	
Gap	36.5***		105.3***	1.5		76.8***	
**Conversation**							
Boys	1.0		6.3	11.4		12.7	73.33
Girl	2.6		12.8	17.4		24.2	82.32
Gap	−1.6		−6.5***	−6.0***		−11.5***	−8.98***
**Nothing**							
Boys	12.8	29.3	16.5	13.6		7.6	31.72
Girl	15.5	33.1	16.7	11.2		6.6	34.72
Gap	−2.7	−3.8	−0.3	2.3		0.9**	−2.99***

* *p* < .1.

** *p* < .05.

*** *p* < .01.

**TABLE A5:** Tobit estimates of gender on leisure activities by country.

	Belgium	United Kingdom	United States	Japan	South Korea	South Africa	India
General leisure	−5.74 (13.97)	−72.07*** (11.47)	−51.85*** (4.58)	−32.69*** (2.22)	−47.72*** (3.94)	−10.47 (7.51)	−42.18*** (3.62)
Sports/Exercise	−30.78*** (8.01)	−21.35*** (5.18)	−30.23*** (3.99)	−51.99*** (1.57)	−14.87*** (1.36)	−30.75*** (3.12)	−5.87*** (0.87)
TV/Radio	−1.99 (9.32)	18.23* (9.30)	−6.01*** (0.21)	2.11 (1.50)	14.46*** (3.19)	−27.98*** (6.94)	−0.78 (1.34)
Computer/internet use	−33.02*** (8.28)	−100.24*** (8.56)	5.30*** (0.60)	N/A	−71.23*** (2.99)	N/A	N/A
Conversation	2.13** (0.86)	6.64*** (1.83)	4.61*** (1.36)	N/A	11.17*** (0.96)	N/A	8.68*** (2.08)
Observations	536	776	2198	32,759	4308	1574	15,589

* *p* < .1.

** *p* < .05.

*** *p* < .01.

**TABLE A6:** Average minutes in weekend leisure activities in India by gender: Additional controls.

	Baseline	State dummy	Religion and Caste controls
General leisure: girls	−43.00*** (3.70)	−44.82*** (3.67)	−43.30*** (3.67)
*N*	15,589	15,589	15,589
*R*‐squared	0.02	0.06	0.03
Sports: girls	−6.76*** (1.05)	−7.01*** (1.01)	−6.76*** (1.04)
*N*	15,589	15,589	15,589
*R*‐squared	0.02	0.04	0.02
TV: girls	0.76 (1.44)	0.14 (1.40)	0.91 (1.47)
*N*	15,589	15,589	15,589
*R*‐squared	0.05	0.11	0.05
Conversation: girls	8.35*** (2.25)	7.87*** (2.14)	8.23*** (2.22)
*N*	15,589	15,589	15,589
*R*‐squared	0.05	0.07	0.05

*Note*: India Time Use Survey, 2024. Level of significance: **p* < .1, ***p* < .05, ****p* < .01. Column 2 includes state dummies, and Column 3 includes religion (base: Hindu and dummy variables for Muslim and Others) and caste categories (base: Scheduled Tribe, dummies for Scheduled Caste, Other Backward Classes, Others). Robust standard errors are clustered at the state level.

**TABLE A7:** Interaction coefficients of gender and age across countries.

	BE	UK	US	JP	KR	ZA	IN
General leisure	−17.78** (6.67)	−8.09* (4.85)	−5.36 (0.60)	−5.25** (0.93)	−9.11*** (1.78)	−4.59 (3.50)	−9.40** (1.60)
Sports/exercise	2.59 (3.99)	−1.13 (2.20)	6.17 (5.05)	3.67*** (0.66)	2.04*** (0.68)	−1.52 (1.62)	−1.16** (0.22)
TV/Radio	1.15 (4.19)	−0.08 (4.24)	−0.58 (0.94)	−0.36 (0.63)	−0.60 (1.42)	−4.22 (3.08)	−1.46** (0.57)
Computer/internet use	−9.59** (4.03)	0.99 (3.73)	−4.68 (3.93)		−8.33*** (1.35)		
Conversation	−0.37 (0.53)	0.34 (0.67)	−3.72 (0.15)		0.52 (0.38)		0.24 (0.85)

*Note*: Standard errors in parentheses.

* *p* < .1.

** *p* < .05.

*** *p* < .01.
